# Supplementary material for: DMRforPairs: identifying Differentially Methylated Regions between unique samples using array based methylation profiles
Source: BMC Bioinformatics. 2014 May 15;15:141. doi: 10.1186/1471-2105-15-141 (PMC4046028; doi:10.1186/1471-2105-15-141)
Supplement: Additional file 2 — DMRforPairs output for the comparison of A431-MCF7 and NA17018-NA17105. Please start from the HTML files in each folder. Available via the BMC Bioinformatics website. [file 1471-2105-15-141-S2.zip › 1394847754114233_MOESM2_ESM/A431_MCF7/figures/33136.pdf]

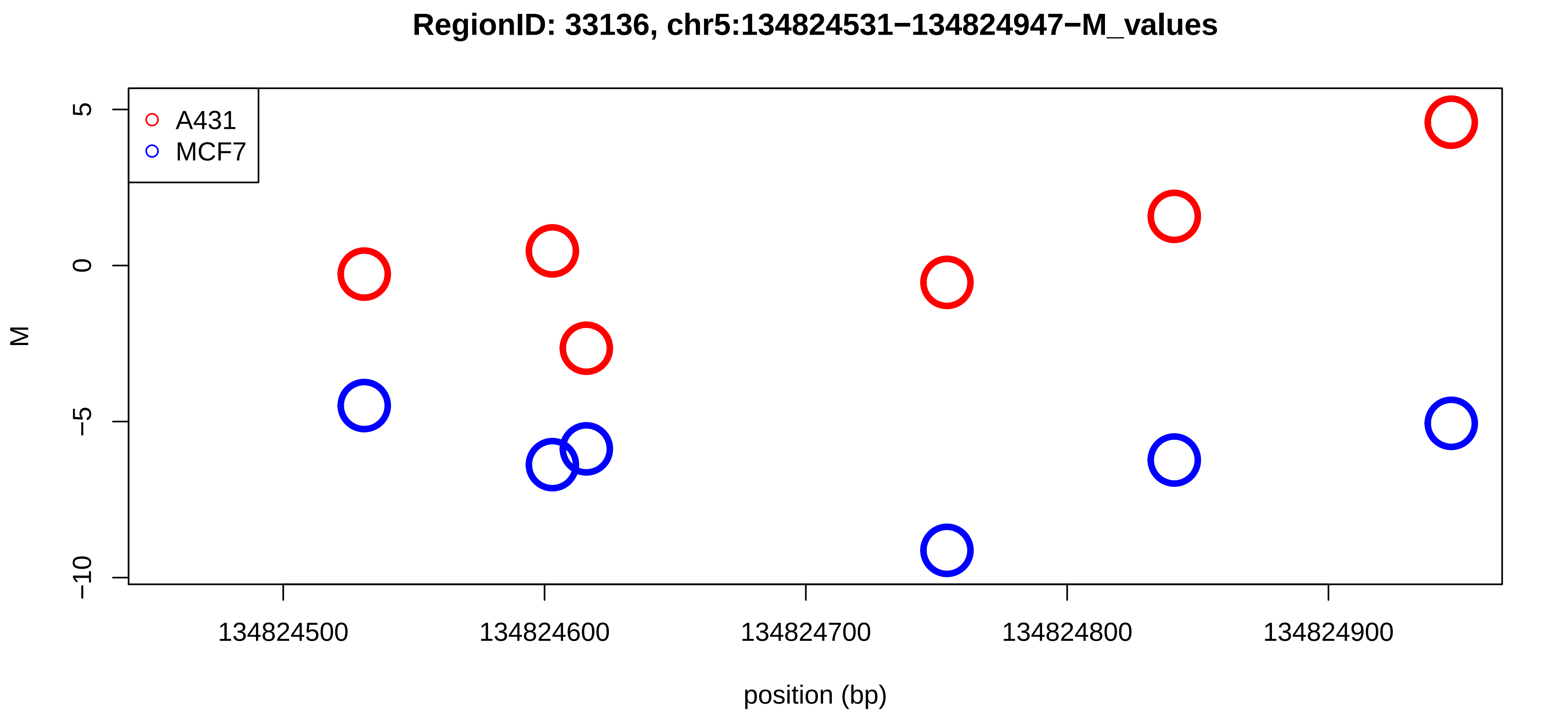

RegionID: 33136, chr5:134824531−134824947−M_values
A431
MCF7
M
5
0
−5
−10
134824500
134824600
134824700
134824800
134824900
position (bp)

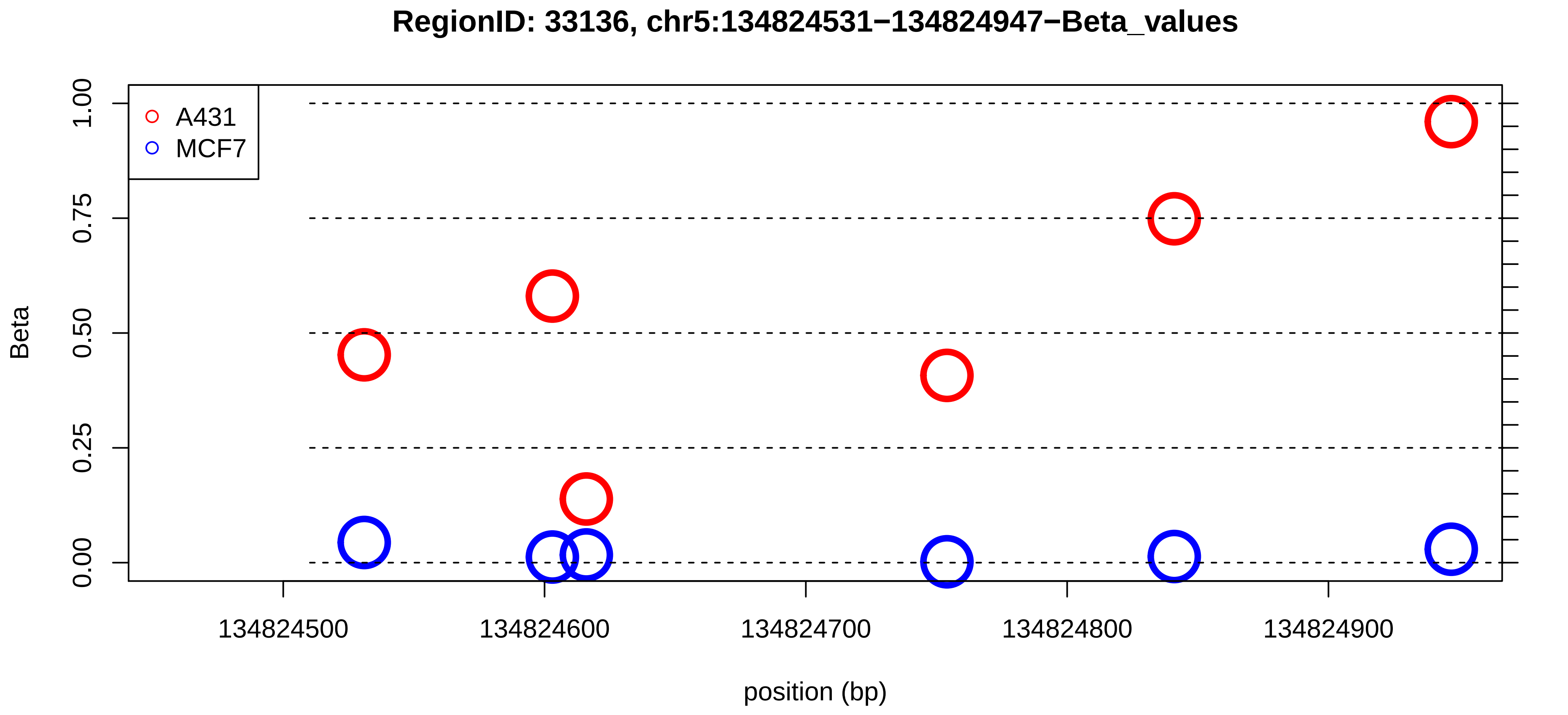

RegionID: 33136, chr5:134824531−134824947−Beta_values
A431
MCF7
Beta
1.00
0.75
0.50
0.25
0.00
134824500
134824600
134824700
134824800
134824900
position (bp)

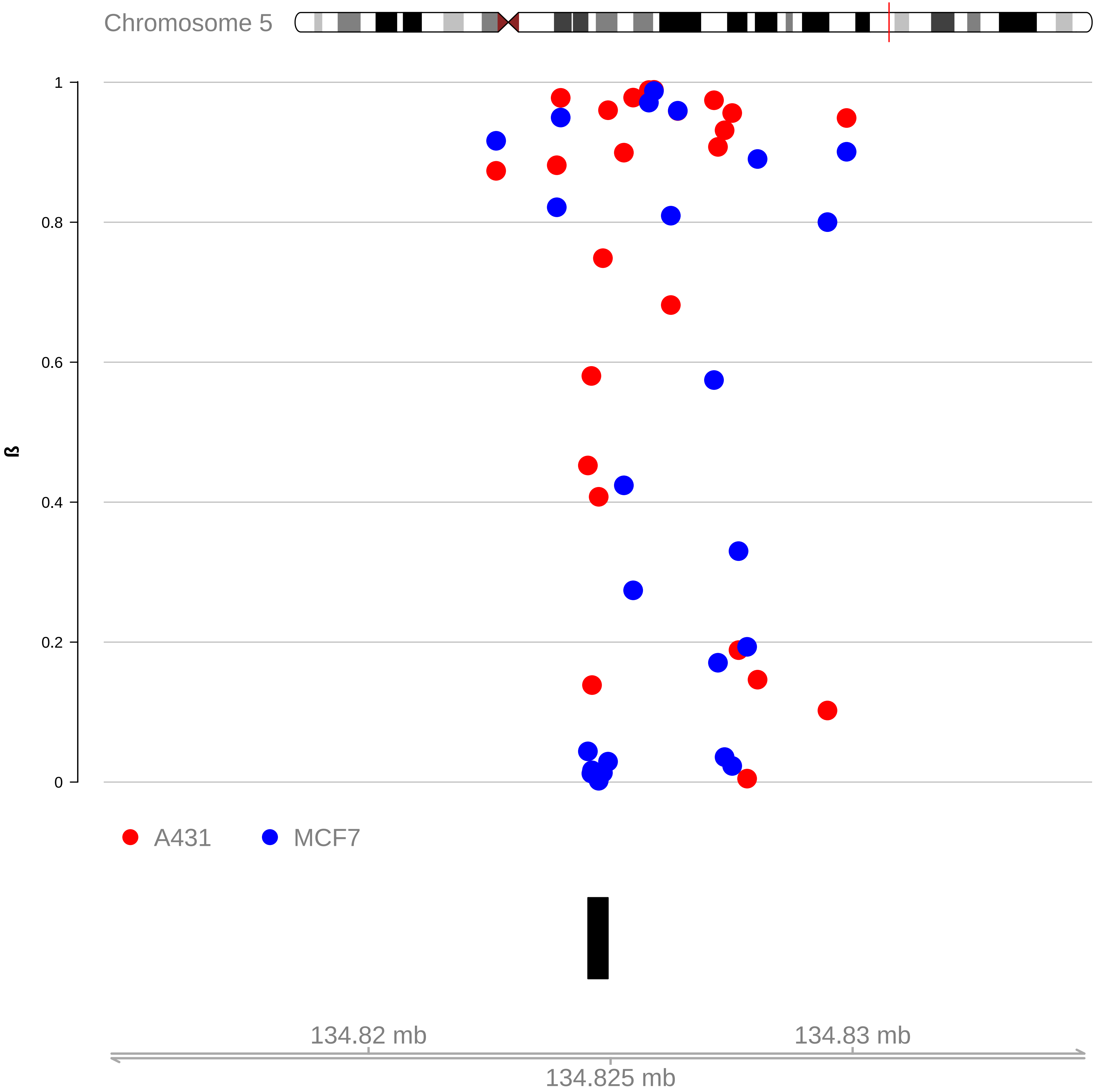

Chromosome 5
1
0.8
0.6
0.4
0.2
0
ß
A431
MCF7
134.82 mb
134.83 mb
134.825 mb
